# Medical and Pathological Observations, Drawn from Military Hospital Practice, with Cases, &c.

**Published:** 1818-03

**Authors:** Richard Webster

**Affiliations:** Surgeon of the 51st Regiment


### Journal and Review.

VOL. V.] MARCH, 1818. [NO. 27.

### PART I.

### ORIGINAL COMMUNICATIONS.

——— quid utile ———

*Medical and Pathological Observations, drawn from Military Hospital Practice, with Cases, &c.* By RICHARD WEBSTER, Esq. Surgeon of the 51st Regiment.

THOSE who have had opportunities of witnessing the medical practice of the army, and that of civil life, must have observed the very great difference which exists in the type of the diseases of the soldier and the civilian.

The habits of the former appear to dispose him to an inflammatory diathesis; he is, generally speaking, of an age at which diseases of excitement occur; he is well clothed and fed; has regular, and, in general, not too severe exercise; he is free from care, his wants being anticipated; he pines not, like the labourer, at approaching want, nor breathes the tainted air in which the artizan or mechanic spends his days. He knows that, should he be attacked by sickness, every possible comfort awaits him; and it is astonishing how soon he recovers from the effects of privation and fatigue, by returning to his accustomed habits. It appears as if the quantity of food that he is allowed, is sufficient to keep up a proper balance of the circulation, and that its quality is not of a description to produce indigestion; hence one would infer, that there is more health to be found amongst a certain number of soldiers, than amongst an equal number of men of any other description. This I believe to be really the case, under tolerably favourable circumstances; but there are many things in a soldier's life which tend to throw him into a state of disease; the principal of which, perhaps, are, intemperance, fatigue, and exposure to the weather under every variation of temperature.

It is not therefore surprising, that the diseases of the soldier, when acute, should be accompanied with a high degree of excitement; and that, when he lingers under *chronic* diseases, they should be, in most instances, the effects of previous *inflammatory* ones.

A review of the diseases of the men in the general hospitals of a large army will, I am confident, justify the truth of the above observations. The physician there justly complains, that he has little left to do but support the sinking frame of the man whose viscera have been destroyed by previous congestion or inflammation.

That long and fatiguing marches, want of proper food, bad clothing, and broken rest, will cause a susceptibility in the human constitution to disease, I am well aware; and that, if those causes are continued, fevers of a low type will be induced. I am well aware, also, that if men, who have been subject to the above causes, be placed in certain situations, many of them will, undoubtedly, have low fever; but I am by no means prepared to admit, that they will have *typhus* under every circumstance. On the contrary, I am convinced, that they will *not*. I believe also, that the type of the disease will be dependent, in a great measure, on the situation and circumstances under which they happened to be placed; and that, although an unusual degree of sickness should prevail, it is not necessary to attribute it to the power of contagion.

After the retreat from Burgos, in the year 1812, the regiment to which I belong came into winter quarters, on the 5th of December.

The men had suffered much from privation and fatigue; they had an emaciated worn-out appearance, and the weather was wet and cold. Their diseases, at this time, as might be expected, were not those of high excitement.

The village, in which we were situated, was at the foot of the Estrella. Every possible attention was paid to the men, and every exertion made to render them as comfortable as possible. Their diet was good and regular, and they had but little duty. This state, so opposite to what they had been lately accustomed to, could not fail to make a great alteration in their appearance and constitutions; but another important change took place, which tended, I conceive, more than any other circumstance, to determine the character of the diseases. A sharp frost set in, with an easterly wind blowing directly over the top of the mountain covered with snow, upon our village, and continued several weeks. The atmosphere was clear; there was scarcely a cloud to be seen between the earth and "the blue ethereal sky;" the air, during the greater part of the day, was sharp and bracing; the nights were excessively cold.

Previous to this change of the weather, and before regular diet, &c. could have much effect upon the constitution, diseases of high excitement did not occur. As soon, however, as a visible alteration took place in the appearance of the men, there was a change also in the character of their diseases; but when the frost set in, and they were exposed to the weather I have mentioned, the symptoms of the prevailing diseases were so unequivocally marked, as to rob me of all credit for discovering their nature.

My chief reliance was upon venæsection, purgatives, and mercury. My loss was trifling, compared with that which was the result of an opposite mode of treatment. In making this comparison, I disclaim any motive, except that of bringing it before the eyes of those who, at a future period, may be thrown into a similar situation, and in order to shew them that, although diseases may, from particular circumstances, be of a low type, yet that they may be quickly changed to one of an opposite character; and that the practitioner, who allows the dread of *debility* to take possession of his mind, unfits himself for observing the changes which take place, and deprives himself of the power of employing the only remedies at present known, that are capable of suddenly and effectually arresting the progress of diseases of excitement.

The influence of the weather on the human constitution, and the effect that it has in producing inflammatory diseases, are very great, and perhaps not, in every instance, sufficiently attended to. The extremes of heat and cold appear to have the effect of exciting diseases of great vascular action; for the operation of the latter cause, it is probably requisite that the weather should be dry, an opposite state being more favourable to the production of fever of a low type.

I have lately paid a good deal of attention to this subject, and find that the influence of the weather on the character of other diseases is very great; even on those which, of themselves, may be considered of minor importance. I am convinced, from experience, that it will be found that some diseases, which, in mild weather, yield to little more than regimen, shall, during the prevalence of a dry north or east wind, require active depletion. I may instance *hernia humoralis, and recent pseudo-syphilitic complaints,* amongst many others. If then, in those diseases, depletion becomes necessary, owing to a peculiar state of the constitution induced by an evident cause, how much more must it be necessary, in diseases accompanied with febrile action, and whose terminations may be fatal, during the existence of the same cause?

In the months of March and April last, I had several cases of fever which required the most active treatment; and, although I am convinced not only of the propriety but of the absolute necessity of bleeding in febrile affections, yet I committed some errors in not being sufficiently active in my practice. As I have long been a strong advocate for venæsection in febrile diseases, and as I have had numerous opportunities of putting it in practice, I must declare, that in no instance have I had to regret its use. I wish I could make the same observation respecting its omission.

On the 22d of April last, I admitted a strong man, aged 23, into hospital, labouring under pyrexia. He had been attacked the preceding day with rigor. He complained of great pain in his head; was extremely drowsy; his face was flushed; his pulse firm; skin hot; and he had great thirst. I bled him to the extent of twenty-six ounces, gave him a purgative medicine, and had him sponged with cold water. In the evening, the symptoms were less violent; but the purgative not having operated, he took some calomel, and divided doses of the common saline purging mixture. He had seven stools during the night. The next morning, the symptoms continued, and I committed a grand error, in taking blood by cupping-glasses applied to the scarified temples, instead of bleeding him largely from the arm. He felt something relieved by this local bleeding; but the pain in his head continued. The scalp was now shaved, and kept constantly wet with cold vinegar and water during the night; and it was remarkable how great an effect it had in diminishing the pain, and removing the stupor. This effect might have probably been attributed to the other remedies which were used, had it not happened that severe pneumonic symptoms supervened; and that I was obliged to have recourse to great and repeated depletion, in order to subdue them. I think that some practical inferences may be drawn from this case. Had I used general instead of local bleeding, or in conjunction with it, on the second day of this disease, it is probable that I should not have been obliged to have recourse to it on the *sixth*, when the oppression was so great, notwithstanding repeated depletion, that the artery of the right wrist could not be felt, and that of the left but very indistinctly, until several ounces of blood had been abstracted.

In the next place, I do contend, that *pain in the head, with a flushed countenance,* is always a sufficient indication for the use of the lancet, from whatever cause the phænomena may arise. This observation may appear so self-evident to most people, as to render it superfluous; but no great length of time has elapsed since men in *fever, accompanied with those symptoms,* were treated in a very different manner. Another symptom, which, although a very common one, I may be allowed to remark upon; I mean *thirst:* this, when severe, at the commencement of a fever, always points out the propriety of depletion; and it is, I believe, a never-failing symptom in diseases of *great excitement*. If I have ever seen a case of inflammation unaccompanied by this symptom, I have not remarked it; and I conceive it is much more to be depended upon than the state of the pulse, which I have known below 65, during very active inflammation.* But the great practical point to be observed in this case is, I conceive, *the cessation of cephalalgia and stupor, from cold ablution of the bare scalp, at a time that the man was labouring under a disease of excessive excitement,* as was proved by the subsequent pneumonic symptoms. Had the cold ablution been omitted, I have not the slightest doubt but phrenitis would have supervened.†

The deductions from this case lead me to the relation of one of Enteritis, which occurred in the summer of 1815, while the army lay before Paris.

The subject of it was a young man, aged 19; he had

---

* See Dr. Kennedy's very interesting Case of Inflammation of the Psoac Muscles, in No. XX. of this Journal. EDIT.

† See some very interesting observations on this subject in the last number of the London Med. and Phys. Journal. p. 104, by Dr. Kinglake, between whom and Mr. Webster, there appears much coincidence of opinion, as will be seen farther on. EDITORS.

been heated by exercise at drill, and, while in a state of violent perspiration, drank a large quantity of cold water. He was instantly seized with pain in his bowels. This happened about seven in the evening. He had two loose stools at midnight. I saw him the next morning, about thirteen hours after the attack. The pain in his abdomen was very acute; his skin was hot and dry; *his thirst intense.* I bled him, and put him in the warm bath: those remedies were several times repeated. He died in seventy hours from the time he drank the water.

On dissection, I found the mischief to be so great, that I naturally doubted the propriety of my practice. I think I can have no reason to conclude but that depletion was proper; but, having frequently asked myself what benefit I could expect from the warm bath? I can find no answer to satisfy my doubts, as to the propriety of the treatment. In most other cases of pyrexia, attended with an increased temperature of the body, we find *cold* ablution or affusion eminently useful; it has the effect of cooling the skin, and lessening the frequency of the pulse: its effects are immediate, or nearly so. In a case like this, which is to terminate in a few hours, it is of the utmost consequence that a vigorous and decided mode of practice should be adopted; for, although the man lived seventy hours, I am confident, that before the expiration of forty, there was sufficient lesion to preclude the possibility of recovery. By the use of the bath, I conceive that his pulse was accelerated at least ten or twelve strokes in the minute; and I am confident, that had I used *cold* ablution, it would have decreased in the same ratio. I am well aware that the warm bath is recommended by most authors in Enteritis; but this does not make me the more easy, or do away with the unpleasant impression which this case has left upon my mind; for I certainly do think, that if I had kept a cloth wet with cold water to his abdomen, and sponged his body generally, that the chance of recovery* would have

* In the last No. of the *Medical & Physical Journal*, Dr. Kinglake relates a case which strikingly corroborates Mr. Webster's suggestions. "An instance of peritonæal inflammation, in a middle-aged female patient, has fallen under my observation, in which the abdomen was more prominently enlarged than is usual, either in an advanced stage of pregnancy, or in that of ascital dropsy. The tumefaction was insufferably painful on pressure; the pulse was small, quick, and hard; the general strength appeared to be giving way, and there was every prospect of approaching gangrene. The medical practitioner informed me that but little more had been done than moving the bowels, and fomenting with *warm water* the swollen abbeen much greater. That practice must be totally inefficient; and consequently, I think, highly improper, when all the membranes of the abdomen are so changed in their appearance, in a few hours, as to exhibit one uniform mass of disease; when the intestines appear to have been in a high state of inflammation; when the omentum is found adhering at all points; when the vessels of the peritonæum are apparently obliterated, and instead of a thin pellucid membrane, it appears like a sheet of thick glazed paper. What can any man say to himself, on observing those appearances, but that his practice has been totally useless; that it has made no impression on the disease; and that he must look for more appropriate remedies to be employed on a similar occasion. The more I reflect on this case, the more I am convinced that I treated it improperly; and should I be so unfortunate as to meet with another of the same nature, I shall certainly have recourse to cold ablution, and keep the abdomen wet with cold vinegar and water.

Would not the repeated application of leeches to the abdomen, so as to keep up a perpetual flow of blood, be useful?

Those who have not practiced the ablution of the shaved head, with cold water in fever, can have no idea of its efficacy in lessening pain. If this is effected by gradually decreasing the temperature of the water, and thereby restraining the increased momentum of the blood, why might not the same effect take place in the intestines, by a similar application to the parietes of the abdomen? In both cases, there is a great communication between the internal blood-vessels and those of the surface; and I see no reason why it should not be useful in one case, as well as in the other, where the pain, heat, and thirst, are as

---

domen. As no benefit had been afforded by warmth, it was proposed to see what might be effected by cold. Cloths wetted in cold vinegar and water were incessantly applied over the whole surface of the distended belly, at short intervals, during some hours, with all the relief that could be desired, and much more than could have been reasonably expected. The patient speedily recovered." P. 105.

Mr. Webster's paper was in our hands before this case of Dr. K. was published, and the coincidence of opinion is very striking. For our own parts, we conceive that the warm bath, in Enteritis, has a very doubtful, if not a hurtful effect, until the inflammatory action is checked by general and local bleeding, together with purgatives. We have seldom used it but where *obstipation* was obstinate, and the excitement greatly reduced by other means.—EDITORS.

great as they were in this instance. It will be observed, that I am not attempting to depreciate the use of the warm bath in Enteritis, generally. I am well convinced that there are many cases, in which it may be highly advantageous; but I wish to suggest the propriety of distinguishing those cases from others, in which it may be prejudicial. In other words, I mean to say, that the warm bath ought not to be employed during a state of great excitement, or because it happens to be called *Enteritis.**

The practitioner who employs venæsection in febrile affection alone, does not avail himself of all the advantages to be derived from remedies in many other diseases. Fresh proofs are brought forward every day of the efficacy of venæsection in diseases which, not long ago, were treated by cordials and stimulants; and we have had people lingering for years under painful and truly distressing complaints, which we have now reason to believe would have yielded to venæsection and low living. It behoves, therefore, every man who has proper opportunities (let his abilities be ever so humble), to contribute his share of practical facts, in order to prove the efficacy of venæsection in spasmodic diseases. Few people, perhaps, have better opportunities than regimental surgeons, in the time of peace, of making observations on diseases, and the effects of remedies; and I am convinced, that the very excellent regulations which have been established by Sir James M'Gregor, for conducting the practice of army hospitals, will be the means

---

* Extract from my half-yearly Return to Sir James M'Gregor, Sept. 20, 1816.—The Editors of the Medico-Chirurgical Journal have lately had opportunities of observing the excellent codes of medical discipline introduced by Sir James M'Gregor, and the able and zealous inspectors now employed, into the military hospitals throughout the kingdom. The symptomatology and pathology of diseases are now delineated at the bed-side, with the most scrupulous exactness and detail. These documents, compared with recoveries, relapses, and *post mortem* appearances, enable the inspectors of hospitals, and even the head of the medical department, to form the most accurate judgment not only of the therapeutics, but of the whole scope of genius, knowledge, and talent of the practitioner. The vast utility of this measure is at once apparent. Ability will be developed and fostered—supineness stimulated—incapacity detected! Had such measures been pursued by the army and navy during the long and arduous contest in which we have been engaged, what a vast fund of professional information would have been amassed! what a number of most important facts would have been preserved from the gulph of oblivion! We hope that the present enlightened head of military medicine and surgery, will not allow such depositories of facts as the reports alluded to, to remain unproductive of utility to the medical world at large.—Edit.

of eleciting and preserving many valuable facts; and as the cases are daily registered, the Reporters of them will not be likely to fall under that censure of Dr. Cullen, when he observed that, "*there were more false facts than false theories.*"

On the night of the 27th of March, 1817, Patrick Reynolds, aged 26, was attacked with severe pain in his abdomen, which recurred at intervals, and for which a dose of sulphat of magnesia had been given to him. I saw him the next morning; the spasms were excessively strong; he had vomited several times; his pulse was 76. By the abstraction of twenty-four ounces of blood, he was reduced nearly to a state of syncope. He then took three grains of the submuriate of mercury and *half a grain of opium.* Immediately after the abstraction of the blood, and before the opium had time to dissolve in his stomach, he fell asleep; the pain ceased, and did not return. He had three stools during the day, each containing hardened fæces. He never vomited after the bleeding. He took another dose of calomel at night, and some oleum ricini in the morning, which produced several hard stools.

Thus this man took but half a grain of opium for the cure of a very severe spasmodic disease. I imagine the liver was in a state of congestion; but, at all events, bleeding, in almost all cases of this kind, will be found the safest practice.

Had a large dose of opium been given to this man, it is most probable that the intestines would not have relaxed so soon, nor have been so readily acted upon by the calomel; it is highly probable also, that the worst symptom, vomiting, would not have so immediately ceased: at all events, the whole materia medica could not have effected a more speedy cure.

Lieutenant F. 51st regiment, was attacked with slight pyrexia, attended with swelling of the tonsils and pains in the respiratory muscles. His bowels were costive. In this state, two grains of the tartrite of antimony were given to him; he was in bed, when the antimony began to operate as an emetic. He was lying on his right side, with his head stretched over the side of the bed; consequently, the muscles of the left side of the abdomen were in some degree on the stretch;—the vomiting was very severe; he strained greatly for near half an hour. Shortly after this, he had frequent pains in the muscles of his chest and abdomen. Several days afterward I saw him; he was complaining of severe shooting pains in different parts of his body, which were worse at night. His tongue was white; pulse variable, both as to strength and frequency; he could not take the least exercise, without feeling extremely weak and exhausted; he had frequent partial perspirations and scarcely any appetite. Conceiving his complaints to be, in a great measure, dependant on the state of the chylopoetic viscera, I directed him some purgative, and to live low. The pains increasing, I directed him the warm bath; this, however, appearing to do him harm, was soon discontinued. The pains became more severe; the perspirations more profuse: one day he fainted. He was now generally attacked about midnight, with the most dreadful spasms, which usually lasted about four hours. Opium and æther had but little effect in affording him relief; and, although a man of great fortitude and bravery, his resolution was broken by the excess of pain which he suffered, and the house in which he lodged was, much against his wish, disturbed by the noise he made during the continuance of the paroxysm. He was growing thin; the colour had left his cheeks, and his eyes were sunk: his appearance altogether was much the reverse of what it had been a short time before. The regularity with which the spasms returned, at a certain period, induced me to try the arsenical solution. This he took two days, but without any further effect than to alter the time of the attack, on the second night, until four in the morning. The perspiration, during the paroxysms, was so great, that not only the bed-cloathes, but the bed itself required drying in the morning. In this state I bled him, directed him to take no animal food, and to drink nothing but water. The operation was performed at eight in the evening: *that night he slept well without opium, having scarcely any pain.* I now availed myself of the advice of my friend Mr. Hennen, Deputy Inspector of Hospitals; and we agreed that the bleeding should be repeated, and that leeches should be applied to his chest. *This was done, and he had no more spasms.* The first blood was buffed; the second more so. He took purgatives, composed of calomel and scammony, every second day; and was becoming convalescent, when he used too much walking exercise during the fair in Portsmouth, and has had a slight relapse, but is again recovering, by one bleeding and the daily use of the mercurial pill.

This case, I think, needs no comment; henceforth I shall look upon the lancet as a most powerful antispasmodic.* I could furnish several cases of severe colic, cured by bleeding to syncope, with large clysters, thrown up before recovery from this state. This plan never fails of producing a copious evacuation. I discharged a man from hospital yesterday, who was cured by these means. He was attacked with severe pain at two o'clock, P. M. on Saturday last;—he had frequent vomiting for several hours after the attack. He was relieved by *pressure;* but, at the time he arrived at the hospital, which was twenty hours after the commencement of the disease, he could not bear the slightest touch on the abdomen without experiencing most excruciating pain. An insufficient quantity of blood had been taken from him a short time before I saw him. I bled him until he fainted, and had an enema ready, which was thrown up, and which brought away a considerable quantity of hardened fæces. I was obliged to bleed again in the evening. *He vomited but once after the large bleeding,* and retained pills of calomel and colocynth. Before this, he could retain nothing on his stomach.

RICHARD WEBSTER, Surgeon,
51st. Reg. L. B.

Portsmouth, August 1, 1817.

The Editors will thankfully receive Communications of the above description from the medical officers of the army and navy.

* We beg to direct the attention of the medical world to the very interesting paper of Dr. Moulson of Chester, published in the third volume of this Journal, on the subject of bleeding in spasmodic diseases. Also to Dr. Watts's cases of cholera, asthma, &c. In the *mort de chien* of India, and in tetanus, bleeding has often proved useful.—EDIT.

